#  Searching Eyes: Privacy, the State, and Disease Surveillance in America

**DOI:** 10.3201/eid1411.081034

**Published:** 2008-11

**Authors:** Kathleen F. Gensheimer

**Affiliations:** Maine Department of Health and Human Services, Augusta, Maine, USA

**Keywords:** Disease surveillance, public health, immunization, book review

Searching Eyes is a history of privacy, a value central to the American democratic way of life, and disease surveillance, a core activity critical to the public health mission of intervening as appropriate to protect the populace from preventable causes of illness and death. Public health surveillance is framed as a social practice that is embedded within particular contexts rather than as a purely technical undertaking insulated from politics, law, economics, ethics, and societal forces. The authors cite encounters with tuberculosis (TB), syphilis, HIV/AIDS, and immunization registry efforts to illustrate the pervasive tension in disease surveillance activities that has existed between privacy and the welfare of society since the inception of surveillance in the 19th century.

Although public health officials take for granted the long-established disease surveillance system that enables them to monitor the public’s health, such practices are not viewed as positively by the populace who contest the feared intrusion into what is perceived as an American’s right to privacy. Even the medical community, our strongest ally in public health activities, has feared the intrusion and encroachment of the doctor–patient relationship. The well-publicized AIDS struggle in the early 1980s captured our attention as we strived to respond to the political and ethical questions that up until that time, had only received an episodic focus.

One drawback of the book is that the authors cover a wide gamut of topics. Consequently, disease-specific topics are presented without adequately providing the necessary background on the disease entity to the reader. Not all readers may fully understand the necessary disease-specific background that would make the discussions of public health intervention understandable. For instance, the chapter on TB presents a history of the disease from the turn of the 20th century. But if the reader were unaware of the public health threat posed by the undiagnosed or nonadherent TB patient, the reader would understandably question the authority of public health practitioners to occasionally take extraordinary steps to ensure that infection is not transmitted within the community.

Even without a comprehensive background on specific diseases, this book will interest a wide audience, not only public health practitioners but the medical and legal community with whom we partner. Searching Eye tackles a topic that deserves more of our respective attention, for as noted by the authors, “The vitality of democratic communities necessitates an ongoing effort to negotiate and renegotiate the boundaries between privacy, society’s limiting principle, and public health, which at its best has sought to expand the role of government as a guardian against disease and suffering.” I congratulate the authors on their well-researched and thorough discourse on this core public health activity.

